# Supplementary material for: Human Respiratory Syncytial Virus Infection in a Human T Cell Line Is Hampered at Multiple Steps
Source: Viruses. 2021 Feb 2;13(2):231. doi: 10.3390/v13020231 (PMC7913106; doi:10.3390/v13020231)
Supplement: Supplementary file 1 [file viruses-13-00231-s001.pdf]

Supplementary Figure 1

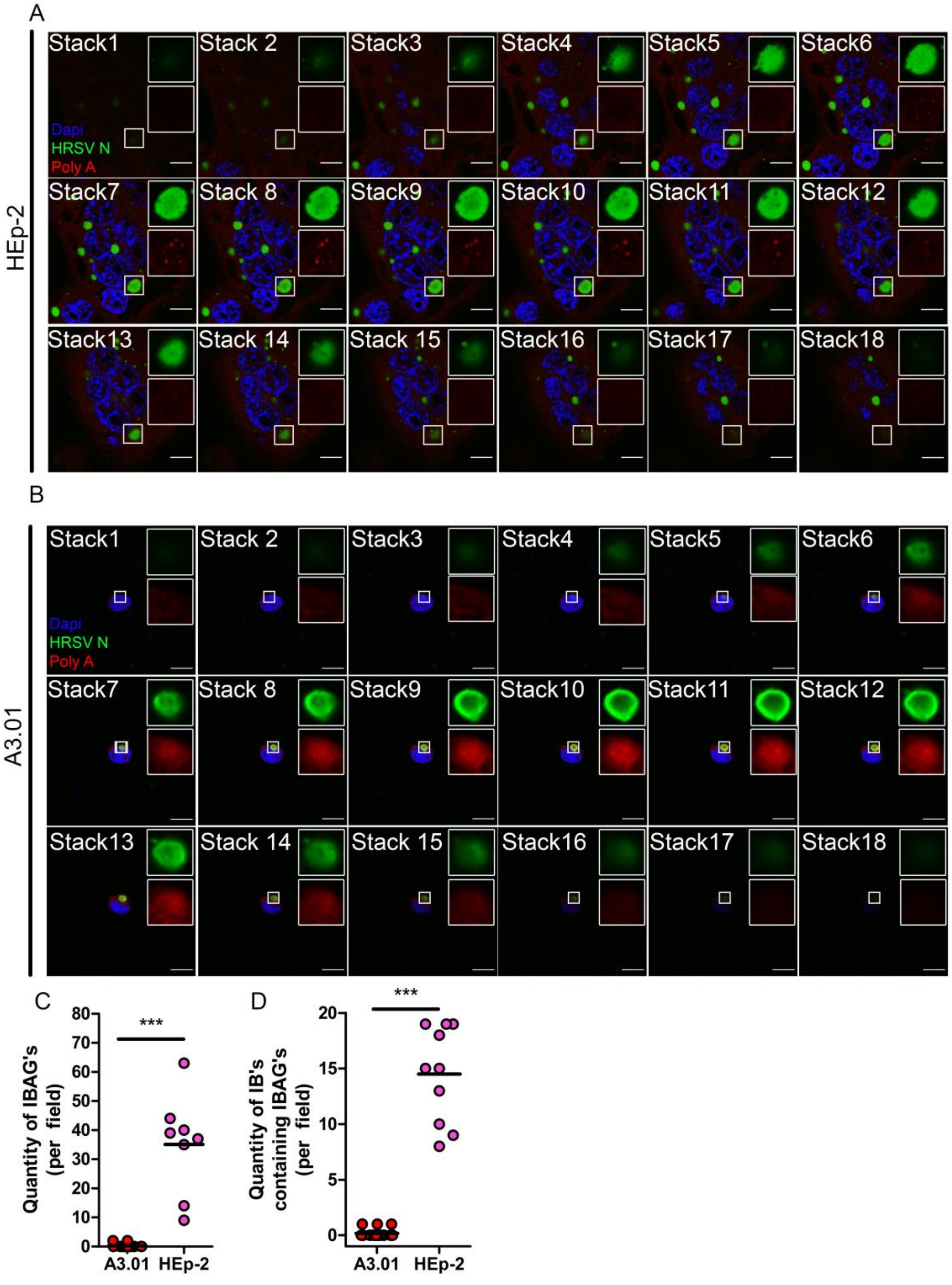

Supplementary Figure 1. Sectional slices from HEp-2 and A3.01-infected cells (supplementary). A, a Z stack was performed, and it is possible to observe IBAGs within HRSV in HEp-2 IBs along with the stacks, highlighted in the crops. B, sequential sectional slices from A3.01 cells, showing IB and the absence of clear IBAGs within, highlighted in the crops. (C) graph of the quantity of the Inclusion Bodies containing IBAGs found in A3.01 and HEp-2-infected cells at 48 hpi. The images were acquired in a Zeiss 780 Confocal, and

are representative of three independent experiments. (D) graph of the quantity of IBAGs counted in inclusion bodies in A3.01 and HEp-2-infected cells. Magnification 63x. Scale bars = 10μm. To the (C) and (D) graphs was counted at least 10 fields of three independent experiments from single focal plane of Z-stacks imaging acquisition. The statistical method used was Student´s T-test, p*<0.05, p**< 0.01, and p***< 0.001.

Supplementary Figure 2

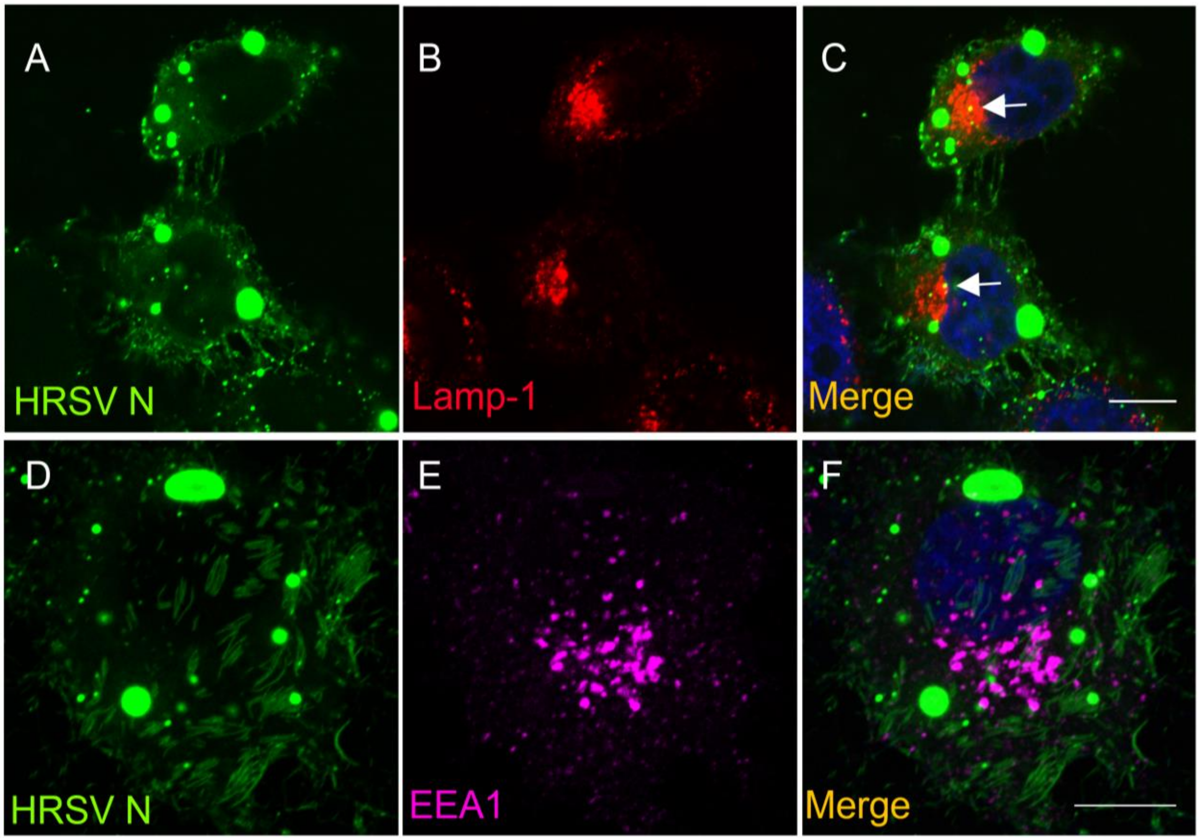

Supplementary figure 2. Co-localization of the HRSV N protein with Lamp-1 and EEA1 in HEp-2 cells. (A-F), HEp-2 cells, were infected with HRSV, and 48 hpi the cells were subjected to immunofluorescence. (A-C) co-localization of HRSV N (A) in green, Lamp-1 (B) in red, and the merge (C). Arrows point to the co-localizations. (D-F), co-localization between HRSV N (D) in green, EEA1 (E) in magenta, and the merge (F). The images were acquired in a Zeiss 780 Confocal, and are representative of a single focal plane of three independent experiments. Scale bar =10 μm.
